# Visual image perception preservation through a compression-encryption framework

**DOI:** 10.1038/s41598-026-45106-y

**Published:** 2026-08-26

**Authors:** Marwa E. Madkour, Salah E. Soliman, Moawad. I. Dessouky, Fathi E. Abd El-Samie, Amir S. Elsafrawey, Mohammed E. Hammad

**Affiliations:** 1 Nuclear Fuel Technology Department, Nuclear Fuel Management Division, Hot Labs Center, Egyptian Atomic Energy Authority, Cairo, Egypt; 2 Department of Electronics and Electrical Communications Engineering, Faculty of Electronic Engineering, Menoufia University, Menouf, Egypt; 3 Engineering Department, Nuclear Research Center, Egyptian Atomic Energy Authority, Inshas , Egypt

**Keywords:** Block Compressive Sensing (BCS), DNA Encoding, Bit-level XOR, Data Mapping, Engineering, Mathematics and computing, Physics

## Abstract

Wireless Sensor Networks (WSNs) are increasingly deployed for monitoring both one-dimensional (1D) and two-dimensional (2D) environmental phenomena, generating vast amounts of sensitive data, often in the form of images, which are transmitted daily. Ensuring secure data transmission over untrusted communication channels is a persistent and critical challenge. Compressive Sensing (CS) has emerged as a powerful signal processing technique that enables simultaneous sampling and compression of signals. Secure Compressive Sensing (Sec-CS) has gained significant attention in information security, as it can serve as an integrated cryptographic mechanism that performs the functions of sampling, compression, and encryption while safeguarding the pseudo-random measurement matrix as a secret key. This paper presents a privacy-preserving, computationally efficient key-agreement framework for secure image exchange in WSN-based monitoring systems. The proposed architecture incorporates DNA encoding, chaotic mapping, and a lightweight XOR-based image encryption operation, all driven by a pseudo-random key vector. The framework not only achieves high computational efficiency but also demonstrates robustness against a range of cryptographic attacks. Extensive numerical simulations validate the proposed framework effectiveness, demonstrating its superiority over existing approaches in terms of both security and computational performance. A comprehensive security analysis further confirms that the proposed framework meets key security requirements, offering strong protection against diverse attack scenarios.

## Introduction

The WSNs operate by transmitting information collected from distributed sensors to a centralized Base Station (BS), where the data can be monitored and analyzed. WSNs have diverse applications, including healthcare monitoring, surveillance, industrial and environmental tracking, process control, performance evaluation, and emergency response in remote healthcare, military, environmental, commercial, and home settings^1,2^. These networks are self-configuring and infrastructure-free, capable of observing environmental variables such as temperature, sound, vibration, pressure, mobility, and pollution. In this study, we focus on Imaging Wireless Sensor Networks (IWSNs), where each sensor node is equipped with a visual sensing unit, making efficient and secure image transmission a critical requirement.

A typical WSN node consists of four main components: sensing, processing, transmitting, and receiving units. The sensing unit captures the required parameter values, but its transmission range is limited due to the low energy capacity of its power supply. Since data is transmitted wirelessly over insecure networks, attackers may intercept sensitive information, making security and energy efficiency primary considerations in WSN design^3–5^.

The CS has emerged as a prominent signal processing technique that enables simultaneous sampling and compression. It is particularly effective for signals that are sparse either in their original form or in a transformation domain. In wireless communications, CS has been widely applied to spectrum sensing, data gathering, channel estimation, and signal recovery^6–10^. To conserve energy, WSNs often use multi-hop routing to transmit data from nodes to the BS. However, the large volume of collected data can create a “hot spot” problem, which can result in bottlenecks or failure points that degrade the overall performance of the network. Compressing the data before transmission is an effective solution, and CS is well-suited for this purpose, as it combines sampling and compression in a single step^11–13^.

This paper presents a secure and efficient visual image compression and encryption framework based on DNA encoding, leveraging the cryptographic advantages of CS and chaotic systems. A significant advantage of employing CS for encryption is that the system does not need to store or transmit the entire measurement matrix; instead, only the pseudo-random generated key or parameters are required. To further strengthen the system integrity, we utilize the high-sensitivity chaotic systems. The proposed framework ensures that only the key stream random generator seed needs to be stored, with updates occurring independently at each end according to an agreed-upon chaotic system.

The pseudo-random number generation is initialized with a seed vector. When both the sensor node and the BS share this seed, they independently generate identical random matrices for encryption and decryption. This dynamic update mechanism, driven by the chaotic map equation, significantly increases security against interceptors while enabling the efficient storage and transmission of large datasets.

The principal contributions of this work are:

- **Integrated Compression–Encryption Framework**: A unified architecture using Block Compressive Sensing (BCS) is introduced for simultaneous image sampling, compression, and encryption, reducing transmission bandwidth while securing data.
- **Dynamic Measurement Matrix Management**: An efficient key agreement strategy based on a modified Tent map updates pseudo-random seeds, enabling both the sensor node and BS to generate identical measurement matrices locally without storing or transmitting large matrices.
- **Multi-Layer Security Protocol**: DNA encoding combined with a bit-level XOR stream cipher ensures high key sensitivity and robustness against statistical, differential, and brute-force attacks.
- **Resource-Efficient Design for WSNs**: Computationally intensive reconstruction tasks ($\:{l}_{1}$ minimization) are offloaded to the BS, minimizing the load on energy-constrained sensor nodes and supporting real-time smart monitoring.
- **Comprehensive Performance Validation**: The proposed framework is rigorously evaluated using chi-square variance, National Institute of Standards and Technology (NIST) randomness tests, and correlation analysis, demonstrating both strong security and superior reconstruction quality compared to contemporary schemes.

## Related works

Recent image compression–encryption schemes can be broadly classified into four categories: (1) CS–based encryption schemes, (2) chaos-based permutation–diffusion schemes, (3) hybrid CS–chaos schemes, and (4) learning-based compressive schemes. In recent years, considerable research efforts have focused on hybridizing CS with chaotic encryption to simultaneously achieve efficient data reduction and strong security guarantees.

Several recent studies demonstrate this trend. For instance, Liu et al. proposed a secure image compression–encryption framework based on a novel hyperchaotic system and two-dimensional CS, reporting enhanced reconstruction accuracy and improved resistance to statistical attacks compared to conventional CS schemes^14^. Similarly, a recent work introduced an efficient compression–encryption scheme combining CS with a four-dimensional hyperchaotic system, achieving good Number of Pixels Change Rate (NPCR) and Unified Average Changing Intensity (UACI) values, while maintaining high transmission efficiency^15^. While these schemes improve cipher randomness and key sensitivity, their reliance on high-dimensional chaotic systems often increases computational complexity and parameter-management overhead, which limits their applicability in resource-constrained WSNs.

1. Comparison with Conventional CS-Based Encryption Schemes

The CS-based encryption schemes exploit the secrecy of the measurement matrix to provide a degree of confidentiality, while achieving compression^7,11,16^. However, many of these approaches require explicit storage or transmission of the measurement matrix or its associated parameters, resulting in increased memory consumption and key-management complexity, particularly in large-scale or distributed WSN deployments.

In contrast, the proposed framework depends on BCS combined with a dynamic chaotic seed update mechanism, enabling both the sensor node and the BS to generate identical measurement matrices locally without exchanging the full matrix. This significantly reduces storage requirements and communication overhead. Furthermore, the use of normalized Gaussian matrices ensures compliance with the Restricted Isometry Property (RIP) condition, leading to a stable reconstruction performance, as evidenced by Peak Signal-to-Noise Ratio (PSNR) values exceeding 30 dB at moderate Compression Ratios (*CRs*).

As with most CS-based schemes, reconstruction quality degrades at low CRs. While the proposed framework maintains acceptable visual fidelity for *CRs* ≥ 0.25, very aggressive compression may not be suitable for applications requiring near-lossless recovery, a limitation shared by recent CS-based schemes.

(2) Comparison with Chaos-Based Image Encryption Schemes

Chaos-based image encryption schemes typically rely on permutation and diffusion operations driven by low- or high-dimensional chaotic maps^17–19^. These schemes exhibit strong key sensitivity and good resistance to statistical attacks; however, they generally do not provide inherent compression, which results in unchanged data volume and higher transmission costs. Recent studies, such as that of Odeh et al.^20^, introduced lightweight Tent-map–based encryption approaches suitable for real-time applications on constrained platforms, but they still lack built-in data reduction capabilities.

The proposed framework overcomes this limitation by tightly integrating compression and encryption into a unified process. DNA encoding and bit-level XOR operations introduce an additional lightweight diffusion layer without imposing significant computational overhead. Experimental analyses, including entropy, correlation coefficients, and chi-square tests, indicate that the encrypted images produced by the proposed framework exhibit randomness characteristics comparable to or exceeding those of recent chaos-only schemes.

Nevertheless, as observed in recent DNA-based encryption approaches, including schemes for secure medical image transmission in healthcare IoT^21^, the proposed scheme remains sensitive to bit errors. In noisy wireless channels, even a single corrupted bit can propagate during DNA decoding, potentially degrading reconstruction quality. This underscores the need for integrating error-resilient channel coding mechanisms in future work to ensure reliable image recovery under adverse transmission conditions.

(3) Comparison with Hybrid CS–Chaos Encryption Schemes

Hybrid CS–chaos schemes represent the most closely-related class of schemes^22–24^. Recent studies combining CS with hyperchaotic systems or adaptive DNA encoding demonstrate enhanced resistance to brute-force and differential attacks^14,15^. However, many of these schemes rely on multiple chaotic control parameters, repeated matrix regeneration, or complex embedding stages^25^, which increases algorithmic complexity and complicates key management.

By contrast, the proposed framework adopts a streamlined key structure consisting of only four control parameters, while still achieving a large key space on the order of 10⁵⁶. The dynamic chaotic seed update mechanism ensures high sensitivity to initial conditions and strong resistance to known-plaintext and related-key attacks. Quantitative comparisons demonstrate that the proposed framework achieves PSNR improvements of approximately 5–7 dB over several recent hybrid schemes at equivalent *CRs*, while maintaining lower computational and communication overhead.

A practical limitation arises from the reliance on floating-point operations in chaotic sequence generation and pseudo-random number initialization. For deployment on low-end sensor nodes lacking floating-point hardware, fixed-point implementations must be carefully designed to preserve chaotic properties, a challenge also encountered by recent hyperchaotic schemes.

(4) Comparison with Learning-Based Compressive Schemes

Deep learning-based CS and reconstruction models have recently shown impressive reconstruction quality, particularly at very low sampling rates^26^. However, they typically require extensive training datasets, high memory consumption, and substantial computational resources, making them less suitable for energy-constrained WSN nodes and real-time deployment scenarios.

In contrast, the proposed framework is model-free and does not rely on offline training. Its security properties can be analytically evaluated using established cryptographic metrics such as entropy, NPCR, UACI, and NIST randomness test results. While learning-based schemes may outperform traditional CS in specific reconstruction scenarios, they often lack formal cryptographic guarantees and introduce additional system complexity.

While several image encryption schemes for WSNs have been proposed, most existing approaches suffer from one or more of the following limitations:

1. **High computational load on sensor nodes**: Many schemes perform complex transformations or iterative optimization directly on energy-constrained sensors, making them unsuitable for real-time monitoring.
2. **Limited integration of compression and encryption**: Traditional schemes often treat compression and encryption as separate stages, leading to increased transmission bandwidth or latency.
3. **Static key management**: Previous works rely on pre-shared keys or large stored matrices, which can be vulnerable to attacks and inefficient for large-scale networks.
4. **Insufficient security evaluation**: Several studies focus only on selective security metrics, without comprehensive statistical, differential, and randomness analyses.

These limitations highlight a clear research gap: the need for a resource-efficient, compression-integrated, and dynamically-secure encryption framework tailored for imaging WSNs. The proposed framework addresses this gap by combining BCS, dynamic measurement matrix generation, DNA-based encryption, and offloading of heavy reconstruction tasks to the BS, ensuring both security and computational efficiency for real-time image transmission.

## Preliminaries

### Block Compressive Sensing

Signal acquisition paradigm of the BCS is accomplished through the same operator as that of CS, but in a block-by-block method. The image is split into $\:\mathit c$ non-overlapping segments, each of dimensions $\:\mathit b\times\:\mathit b$. The CS is applied to each block, independently.

1 $$\mathbf \Phi {\:_{BCS}} = \left[ {\begin{array}{*{20}{c}} {\mathbf\Phi {\:_1}}&{\:0}&{\:0} \\ {\:0}&{\:\mathbf\Phi {\:_2}}&{\: \vdots } \\ {\:0}&{\: \cdots \:}&{\:\mathbf\Phi {\:_c}} \end{array}} \right]$$

For each image segment, compression is fairly identical to the structure for traditional CS. If $\:\mathbf{x}\in\:{\mathbb{R}}^{\mathit {N}}$ represents the sparse signal to be compressed of dimension $\:\mathit \:N=\mathit b\times\mathit b$. Let $\:\left[\mathbf{y}\in\:{\mathbb{R}}^{\mathit {M}}\right]$ be the compressed signal, [$\:\mathbf{\Psi\:}]$ be the projection matrix, and $\:\left[\mathbf {\Phi\:}\right]$ be the measurement matrix. Hence, the signal acquisition becomes as follows:

2 $$\:{\left[ {\mathbf{y}} \right]_{\mathit M \times \:1}}\: = \:{\left[ {\mathbf\Phi \:} \right]_{\mathit M \times \:\mathit N}}{\left[ {\mathbf{x}} \right]_{\mathit N \times \:1}}$$

3 $$\:\mathbf{y}\:=\:\mathbf{\Phi\:}\mathbf{x}=\mathbf{\Phi\:}\mathbf{\Psi\:}\mathbf{k}=\mathbf{\Theta\:}\mathbf{k}$$

The CS concept is “a sparse or compressible signal $\:\mathbf{x}\:$ of dimension $\:\mathit N$ can be transformed into $\:\mathbf{y}$ utilizing $\:\mathit M$ ; where $(\:\mathit M\ll\:\mathit N)$”, as in Eq. (3).

Two conditions for the $\:\mathit M\times\:\mathit N$ matrix $\:\:\mathbf {\Phi\:}\:$ are required to keep the inherent properties; like the energy of $\:\mathbf{x}$ in the transformation step ($\:\mathbf{x}\in\:{\mathbb{R}}^{\mathit{N}}\:\text{ to }\mathbf{y}\in\:{\mathbb{R}}^{\mathit{M}}$), low mutual coherence between columns and a RIP condition. The recovery process includes an underdetermined system since the number of equations is small compared to unknowns. So, there is not a single solution. The availability of reasonably fast reconstruction algorithms is important. Using $\ell_0$-minimization for recovering the signal $\:\mathbf{x}$, we get,

4 $$\:min{\parallel\:\widehat{\mathbf{x}}\parallel\:}_{0}/, s.t.\:\mathbf{y}=\:\mathbf{\Phi\:}\widehat{\mathbf{x}}$$

In^27^, the BCS technique using iterative re-weighting $\ell_0$ norm minimization was proposed, and the necessary signal sparsity was accomplished using dual-tree DWT. The Smoothed Projected Landweber (BCS-SPL) algorithm for BCS reconstruction was performed using bivariate shrinkage for the thresholding step in the Landweber iteration. Wiener filtering was employed as the smoothing operator, based on statistical information estimated from a local neighbourhood of each pixel.

### Pseudo-random measurement matrix generation

The proposed framework employs BCS to reduce computational overhead. Rather than processing the entire image with a single massive operator, the image is partitioned into blocks of size $\:\mathit b\times\:\mathit b$. A compact measurement matrix $\:\mathbf{\Phi\:}$ *;* with (*M×N*); $\:\mathit{ M\:=\:\mathit{CR}\:\times\:\mathit N}$ and $\:\mathit{ N={\mathit b}^{2}}$, is then applied to each block.

#### The seed update mechanism

To eliminate the need for storing or transmitting the full $\:\mathbf{\Phi\:}$ matrix, it is generated locally at both the sensor node and the BS using a dynamic seed and a Pseudo-Random Number Generator (PRNG). This seed is updated via an improved chaotic sequence generation map, based on the modified Tent map^22^.

The BS distributes two initial control parameters, $\:\mathit {u}_{0}$ and $\:\acute{\mathit{z}_{0}}$. To amplify the sensitivity of the sequence generation—ensuring that even microscopic fluctuations in initial values result in a completely different matrix—a multiplier of 10 is applied. The seed is updated according to the following equation:

5 $$\:\mathit{z}_{0}=\:mod\:(\mathrm{cos}(\mathit{u}_{0}\:\times\:\:\mathrm{arcos}\left(\acute{\mathit{z}_{0}}\right))-\:\frac{\acute{\mathit{z}_{0}}^{2}}{3})\times\:10\times\:{2}^{16},1)$$

#### Matrix Construction (MT19937)

Once the improved chaotic map calculates the new seed, it serves as the initialization parameter for the **MT19937** PRNG:

1. **PRNG Initialization**: Initialize MT19937 using the updated seed $\:{\mathit z}_{0}$.
2. **Generation**: Generate a sequence of random numbers to construct the $\text {measurement}$ matrix.
3. **Normalization**: The matrix is formatted as a normalized version of the Gaussian measurement matrix. This ensures that it satisfies the RIP condition, which is a mathematical prerequisite for high-fidelity image reconstruction.

### DNA encoding

The structure of DNA has four nucleic acids, every two are complementary to the remaining two; where A and T are complementary (A is symbolized by “00”, T is symbolized by “11”) and C and G are complementary (C is symbolized by “01” and G is symbolized by “10”). DNA sequence is used to transform the type of image from binary to DNA based on the principle of “complementary base pairing rule”. An 8-bit image can be represented by a DNA sequence of length 4. In this case, the pixel code of “139” (its binary representation) is “10001011” and its DNA representation is “GAGT”. The DNA encoding has many rules according to Table 1^18,19,28,29^. The first rule is used in our simulation, Table 2 illustrates the XOR operation between nucleic acids.

**Table 1:** DNA coding rule.

Rule	1	2	3	4	5	6	7	8
00	A	A	T	T	C	C	G	G
01	C	G	C	G	A	T	A	T
10	G	C	G	C	T	A	T	A
11	T	T	A	A	G	G	C	C

**Table 2:** DNA XOR operation.

*⊕*	A	G	C	T
A	A	T	C	G
G	G	A	T	C
C	C	G	A	T
T	T	C	G	A

### Bit-level XOR stream cipher

A symmetric key-based cryptographic technique is utilized to both encrypt and decrypt data. Using a private key, the keystream generator creates a pseudo-random sequence, or keystream. To construct a ciphertext bit, the created keystream bit is XORed with the plaintext bit. Bit-level XOR is an example of a stream cipher encryption. Using the same key, the original image (plain text) is recovered from the ciphertext during the decryption process. The stream cipher is best suited for low-complexity, high-speed operations. Sharing of the predefined initial control parameters ($\:\mathit {k}_{1},\mathit{k}_{2}$) between sensor nodes and the BS is the BS responsibility^30^. These two control parameters are used to control the keystream generation at both sides.

**Key management and encryption**

Security is managed through a tiered system, where the BS serves as the central authority^23^, responsible for the secure distribution of four primary control parameters as follows:

- **Measurement Keys** $\:{\mathit{u}}_{0},\:\acute{\mathit{z}_{0}}$: used for the BCS measurement matrix seed generation and update.
- **Encryption Keys** $\:{\mathit{k}}_{1},\:{\mathit{k}}_{2}$: used for the subsequent bit-level stream cipher.

After the initial compression and DNA encoding, a second layer of defence is applied through a bit-level XOR stream cipher.

1. **Keystream Generation**: A pseudo-random sequence generator is initialized using the private keys $\:{\mathit{k}}_{1}$ and $\:{\mathit{k}}_{2}$. This generator produces a keystream vector $\:\mathbf{s}$ with a length that equals the exact length of the compressed/encoded data.
2. **Encryption**: The final ciphertext $\:{\mathbf{X}}_{\it{Cipher}}$ is produced by performing a bitwise XOR operation between the encoded data $\:{\mathbf{X}}_{\it{DNA}}\:$ and the keystream $\:\mathbf{s}$.

## Proposed crypto-CS framework

**Fig. 1:**
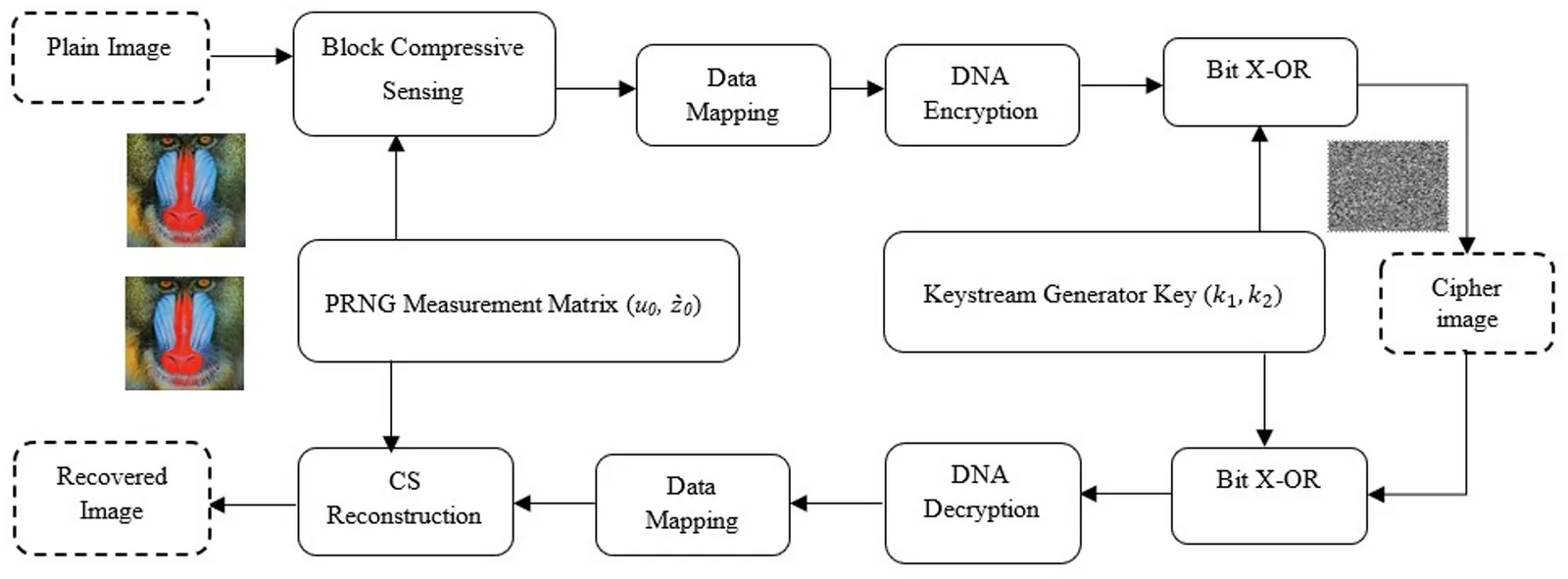
Block diagram of the proposed framework.

The BS transmits a seed for the BCS measurement matrices generation. The pseudo-random number generation technique starts with this seed. If a node and the BS use the same seed values, the same random measurement matrix $\:\mathbf{\Phi\:}$ is generated for encryption and decryption processes. Because chaos refers to non-linear dynamic systems that appear unpredictable, an efficient scheme^30^ was utilized to distribute measurement matrix seed calculation parameters ($\mathit{u}_{0},\:\:\acute{\mathit{z}_{0}}$) to reduce computational complexity and address security problems for CS-based data gathering schemes. Equation 5 is used for seed update. The proposed framework steps are shown in Fig. 1.

**Pseudocode:**
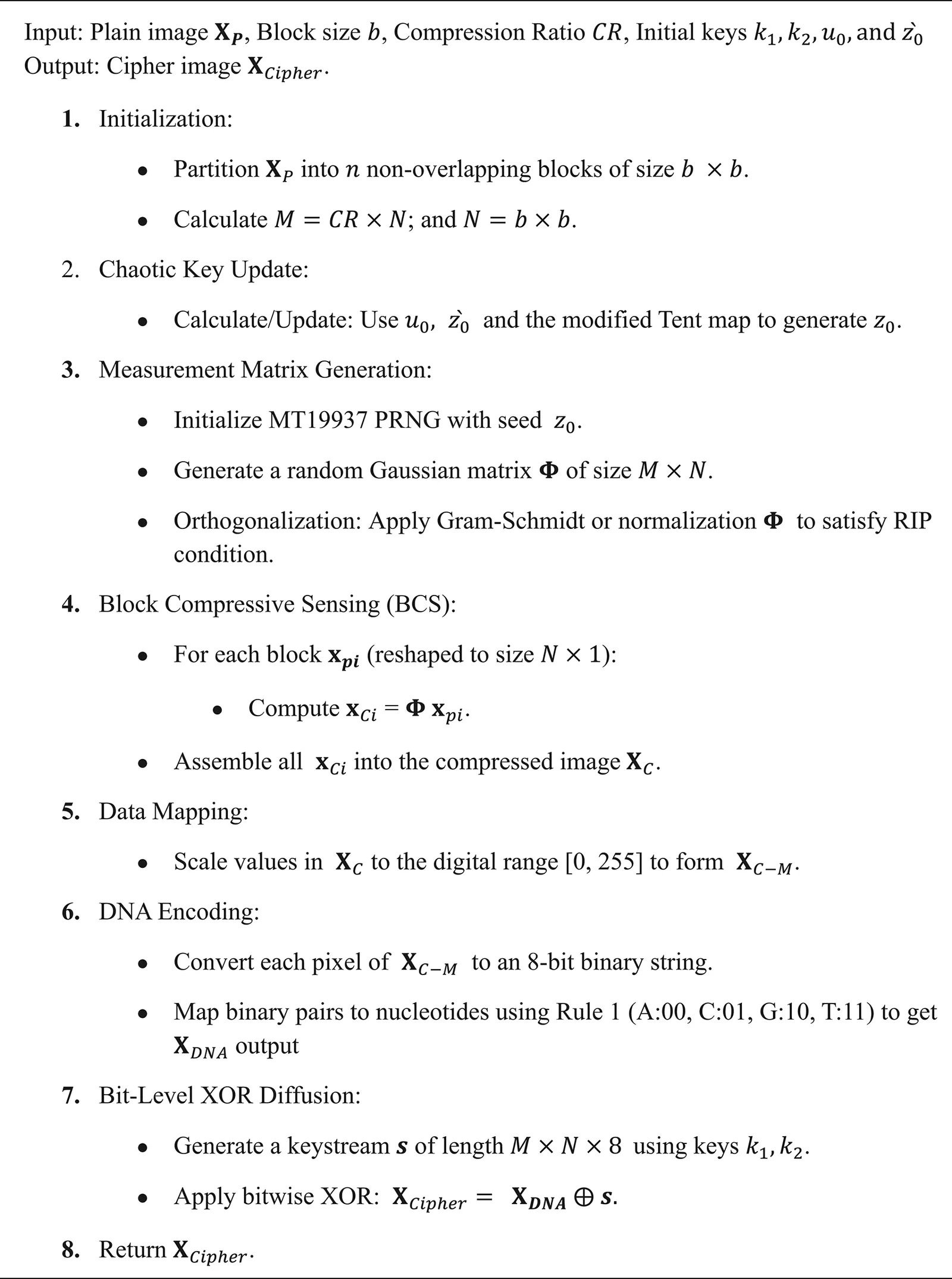
Image Compression-Encryption.

At the receiver end (BS), the process is inverted. The most critical step is the signal reconstruction, which solves the underdetermined system.

**Pseudocode:**
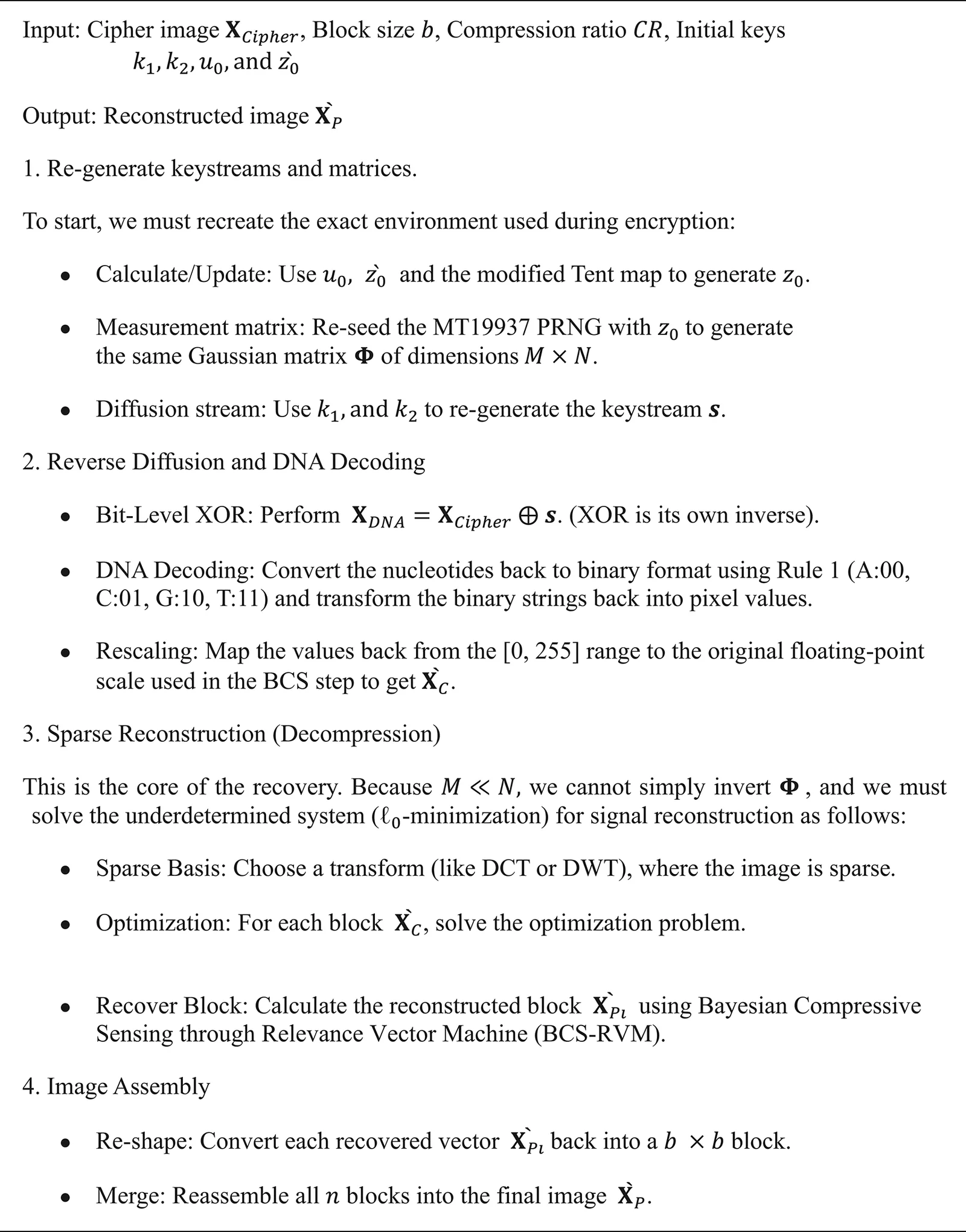
Image decryption-decompression.

## Simulation results and discussion

In this section, we evaluate and analyze the performance and efficiency of the proposed framework using MATLAB R2024a simulations conducted on a system equipped with an AMD Ryzen™ 7 8845HS processor with Radeon™ 780 M Graphics (3.80 GHz), 16 GB RAM (15.3 GB usable), 1 TB storage, and a 64-bit Windows 11 home operating system. The size of the selected test images is 512 × 512 pixels.

The selected images collectively cover a wide range of visual content, ensuring that the proposed compression–encryption framework is thoroughly evaluated across different image characteristics. Specifically, six standard benchmark images from the USC-SIPI image database (https://sipi.usc.edu/database/database.php) are used: Peppers, Baboon, F16, Boat, Tank, and Couple. These images are widely adopted in image processing research and they represent diverse visual features, including smooth regions, highly-textured areas, structured edges, and human shapes.

The measurement matrix $\:\boldsymbol{\Phi\:}$ is generated using a Gaussian random distribution with dimensions (*M × N*) = [(*CR* × *b *× *b*) × (*b *× *b*)]. The block size is set to 32, and the *CR* is fixed at 0.4 for the following experiments unless otherwise stated.

The initial conditions of the chaotic sequence used for seed generation are set to $\:\mathit {u}_{0}=0.3$, and $\:\acute{\mathit{z}_{0}}=0.3$. Furthermore, the control parameters of the keystream random generator used to produce the pseudo-random sequence vector are $\:\mathit {k}_{1}=0.98$, and $\:\mathit {k}_{2}=2.3$.

Our experiments depend on standard reference images for reproducibility and benchmarking. In practical WSN deployments, sensors typically acquire grayscale or RGB images with resolutions ranging from 128 × 128 to 512 × 512 pixels, depending on hardware and energy constraints. The block-based CS and encryption approach is resolution-agnostic, processing images in smaller blocks, which allows efficient compression, encryption, and transmission regardless of the original image size. Thus, the methodology is suitable for real-time image acquisition and secure transmission in resource-constrained sensor networks.

### Performance analysis

For visual image perception, the compression-encryption studies depend on the PSNR performance criterion to examine the decrypted image quality. The reconstructed image quality improves as its PSNR increases. The higher the PSNR is, the less distorted the restored image is. The PSNR is defined as:

6 $$\:\mathrm{P}\mathrm{S}\mathrm{N}\mathrm{R}=20\:{\mathrm{log}}_{10}\frac{255}{\sqrt{MSE}}\:$$

7 $$\:MSE=\frac{1}{{\mathit N}^{2}}\sum\:_{i=1}^{N}\sum\:_{j=1}^{\mathit N}{\left[\mathit{X}\left(\mathit{i},\mathit{j}\right)-\widehat{\mathit{X}}\left(\mathit{i},\mathit{j}\right)\right]}^{2}$$

where $\:\mathit{X}\left(\it{i},\it{j}\right)$ is the pixel value at the $\:\left(\it{i},\it{j}\right)$ position of the original image, and $\:{\widehat{\mathit{X}}}\left(\it{i},\it{j}\right)$ is the pixel value at the $\:\left(\it{i},\it{j}\right)$ position of the restored image. Another metric is the ٍStructural Similarity Index (SSIM). It measures the similarity between the original and restored images, and it is defined as:

8 $$\:\mathrm{SSIM}\left(\mathit X,\widehat{\mathit {X}}\right)=\frac{\left(2{\mu\:}_{\mathit X}{\mu\:}_{\widehat{\mathit{X}}}+{\mathit C}_{1}\right)+\left({2\sigma\:}_{\mathit X\widehat{\mathit{X}}}+{\mathit C}_{2}\right)}{\left({\mu\:}_{\mathit X}^{2}+{\mu\:}_{\widehat{\mathit{X}}}^{2}+{\mathit C}_{1}\right)\left({\sigma\:}_{\mathit X}^{2}+{\sigma\:}_{\widehat{\mathit{X}}}^{2}+{C}_{2}\right)}$$

$\:\text {where }\mathit C_1\:={(\:0.01\:\times\:\:\left({2}^{8}\:-\:1\right))}^{2},\:\text {and }\:\mathit C_2\:={(\:0.03\:\times\:\:\left({2}^{8}\:-\:1\right))}^{2}.$ $\:{\mu\:}_{\mathit X}$ represents the mean value of the original image, and $\:{\mu\:}_{\widehat{\mathit{X}}}$ represents the mean value of the restored image. $\:{\sigma\:}_{\mathit X}^{2}$ is the variance of the original image, $\:{\sigma\:}_{\widehat{\mathit{X}}}^{2}$ is the variance of the restored image, and $\:{\sigma\:}_{\mathit X\widehat{\mathit{X}}}$ is the covariance between the original and the restored images. The structural similarity ranges from 0 to 1. In general, the higher the SSIM values are, the greater the quality of the reconstructed images is. When the original and restored images are identical, the value of SSIM is 1.

**Fig. 2:**
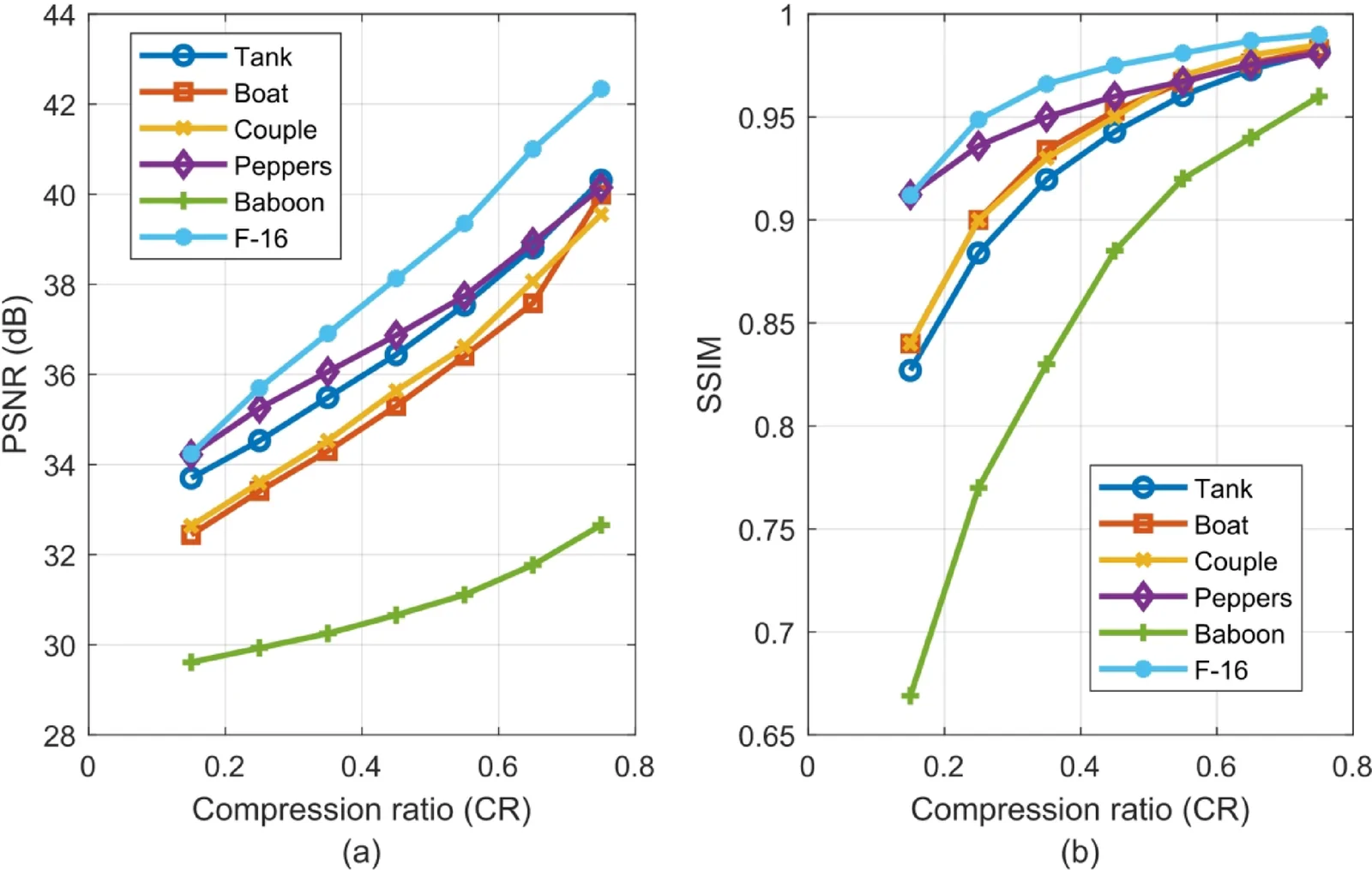
Performance evaluation of the proposed framework with SSIM and PSNR.

For visual assessment, the PSNR and SSIM metrics provide a more effective evaluation. Initially, the performance of the proposed framework is examined using six images of size 512 × 512. The SSIM is used to assess image quality over a range of *CR*s, as illustrated in Fig. 2.b. As the *CR* increases (i.e., as the number of measurements increases), the SSIM value correspondingly improves. Notably, the SSIM remains above 0.8 for all tested *CR*s except for “Baboon”, indicating satisfactory structural fidelity.

In addition, the PSNR is calculated to evaluate the reconstruction performance of the proposed framework under different *CR* values, as shown in Fig. 2.a. The PSNR values consistently exceed 30 dB, which is generally considered a competent level of performance in image recovery tasks. These results demonstrate that the proposed framework achieves high reconstruction accuracy even with a relatively small number of measurements.

### Statistical analysis

1. **Histogram representation**

A histogram is a graphical representation of the distribution of pixel intensities in digital images. When the histogram of an image is non-uniform, statistical information can potentially be exploited by an attacker. If the histogram of a plain image is distinctive and contains noticeable peaks, the system may become vulnerable to statistical attacks. To mitigate this risk, the histogram of the encrypted image should be as uniform and homogeneous as possible.

**Fig. 3:**
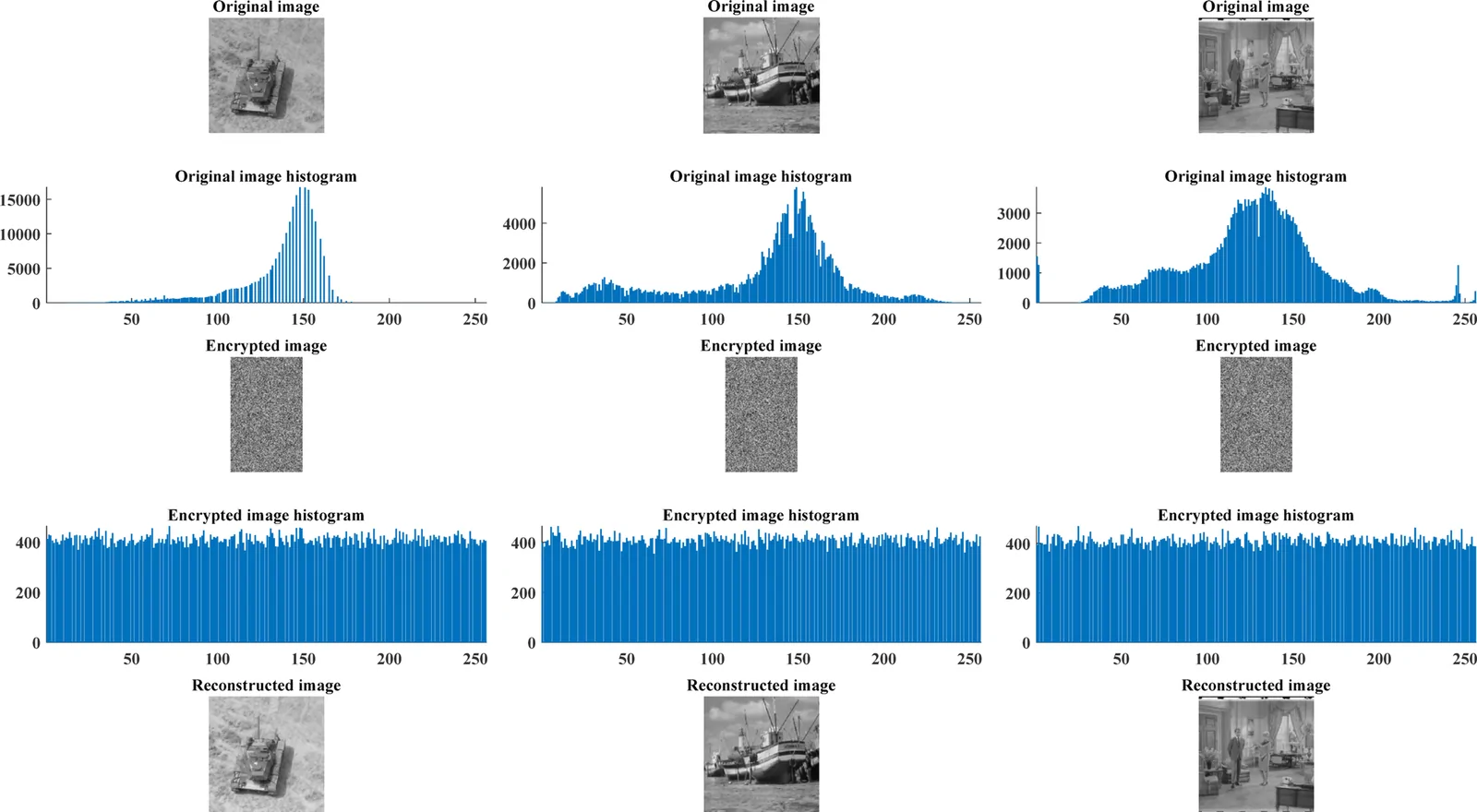
Original image, original image histogram, encrypted image, encrypted image histogram, and reconstructed image.

**Fig. 4:**
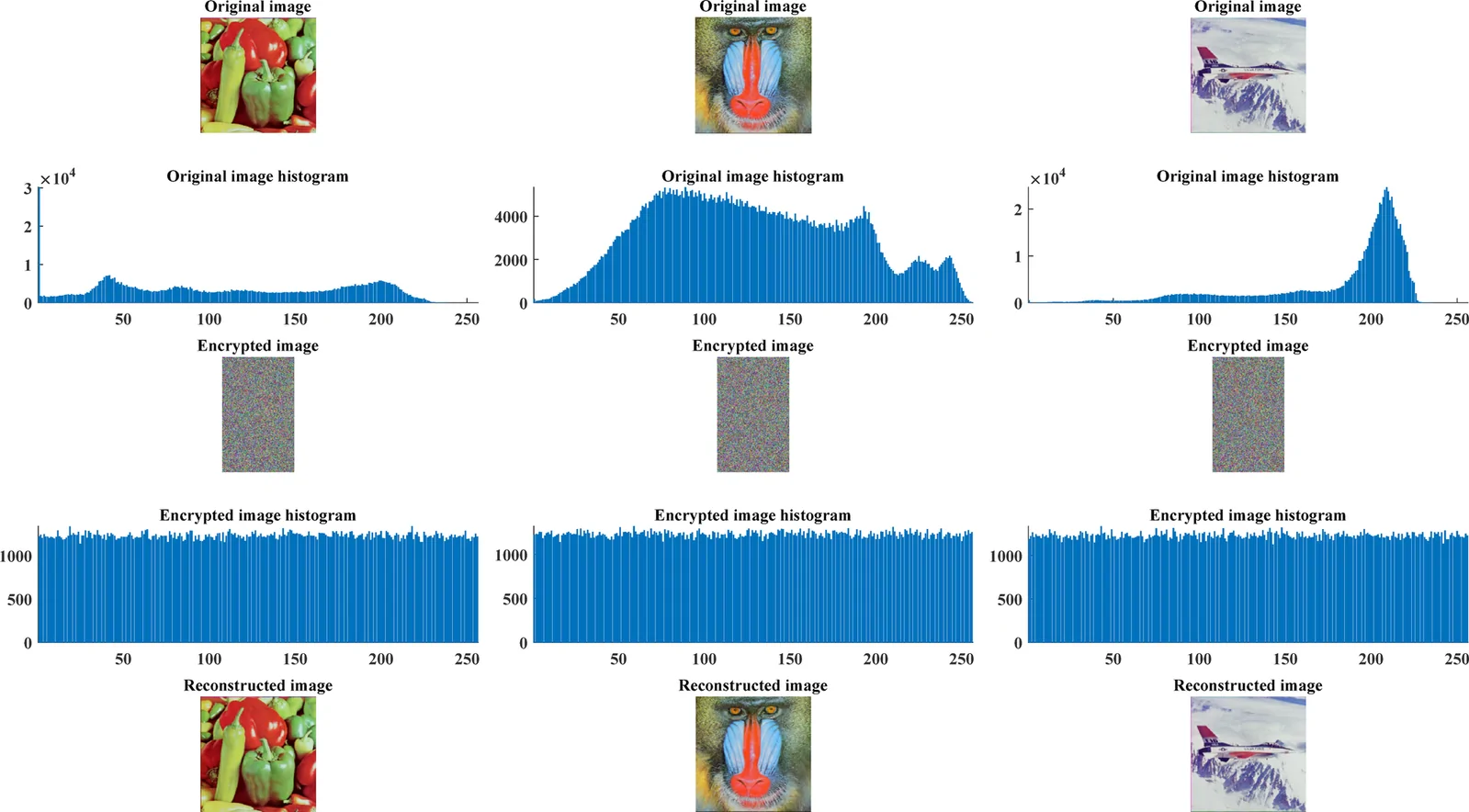
Original image, original image histogram, encrypted image, encrypted image histogram, and reconstructed image.

The encryption results are illustrated in Figs. 3 and 4. The histograms of the original images exhibit clear peaks and non-uniform distributions, which may reveal statistical characteristics to an eavesdropper. In contrast, the histograms of the encrypted images are uniformly distributed and free of noticeable peaks.

These results demonstrate that the proposed encryption framework effectively eliminates the statistical features of the original images, thereby providing strong resistance against statistical attacks.

The visual depiction of the histogram is not always sufficient for measuring the pixel distribution of an encrypted image. The chi-square variance is used as a numerical metric for histogram homogeneity. It is represented as $\:\mathbb {\chi\:}^{2}$. The test compares the observed histogram frequencies of an image to the expected frequencies of a random (e.g., uniform) distribution. Chi-square variance is given by:

9 $$\:\mathbb {\chi\:}^{2}=\sum\:_{\mathit i=1}^{256}\frac{({{\mathit f}_{\mathit i}-\frac{\mathit N^2}{256})}^{2}}{\frac{\mathit N^2}{256}}$$

$\:\mathit {f}_{\mathit i}$ is the frequency of the gray level $\:\mathit i$ . $\:\frac{\mathit N^2}{256}$ represents a completely uniform image, where *N*^*2*^ represents the total count of pixels in the image, assuming a square image. The numerical value of the chi-square variance test at a significant level of 5% is 293.2478^31^.Table 3 shows the value of the chi-square variance test of the proposed framework, which ensures that the values for the proposed framework are not greater than 293.2478. So, the suggested framework is able to resist statistical attacks.

**Table 3:** Results of the chi-square variance test.

Image	Chi-square test $\:\left({\boldsymbol{\chi\:}}^{2}\right)$
Tank	260.8727
Boat	253.7315
Couple	253.6429
Peppers (R/G/B)	234.1951/271.0341/241.9610
Baboon (R/G/B)	239.3463/242.3854/278.9854
F-16 (R/G/B)	239.7024/266.7659/281.9707

In addition, the variance of the histograms of the plain and encrypted images are calculated by:

10 $$\:var\left(\mathit H\right)=\frac{1}{\mathit N^{2}}\sum\:_{\mathit i=1}^{\mathit N}\sum\:_{\mathit j=1}^{\mathit N}\frac{1}{2}{({\mathit {h}}_{\mathit i}-{\mathit {h}}_{\mathit j})}^{2}$$

*\mathit H* is the vector of the histogram values, and *H* = {*h*_1_, *h*_*2*_,...,* h*_256_}. $\:\mathit {h}_{\mathit i}\:\text{ and }\:{\mathit h}_{\mathit j}$ give the numbers of pixels with gray values equal to *\mathit i* and *\mathit j*, respectively. The variance of the histogram is used to measure how spread out the histogram values are. When the variance is low, the image is more uniform. Table 4 gives the values of the variance of the histograms of the plain and encrypted images under test. It can be observed that the variance of the cipher image histogram is significantly reduced compared with that of the plain image histogram. Furthermore, the variance values obtained from the proposed framework histogram results are lower than the corresponding variance values reported in^34^, indicating that the encrypted images produced by proposed framework exhibit more uniform statistical characteristics and enhanced resistance to statistical analysis.

**Table 4:** Results of the variance of the histograms.

Image	Plain image histogram variance	Encrypted image histogram variance (*CR* = 0.4)
Tank	8.1354 × 10^6^	417.8039
Boat	1.5419 × 10^6^	406.3668
Couple	1.2001 × 10^6^	406.2250
Peppers (R/G/B)	8.5609 × 10^5^/1.2785 × 10^6^/1.9734 × 10^6^	376.5490/435.7804/389.0353
Baboon (R/G/B)	3.3266 × 10^5^﻿/5.7347 × 10^5^/3.2102 × 10^5^	384.8314/389.7176/448.5647
F-16 (R/G/B)	2.7243 × 10^6﻿^/2.7407 × 10^6^﻿/4.4488 × 10^6^﻿	385.4039/428.9176/453.3647

2. **Correlation test**

To determine the correlation between two neighboring pixels in the horizontal, vertical, and diagonal directions, the correlation coefficients (*CC*) are calculated. A very low *CC* value (close to 0) indicates that the pixels in the encrypted image are statistically uncorrelated. In contrast, a high *CC* value (close to 1) indicates a strong correlation between adjacent pixels in the plain image. The mathematical expression for the *CC* between two pixels in an image is defined, where $\:\mathit {x}_{\mathit i}$ and $\:\mathit {y}_{\mathit i}\:$ represent the gray values of two adjacent pixels in the image, as follows:

11 $$\:\mathit CC=\frac{\sum\:_{\mathit i=1}^{\mathit N^2}({\mathit x}_{\mathit i}-\stackrel{-}{\mathit x})({\mathit y}_{i}-\stackrel{-}{\mathit y})}{\sqrt{\sum\:_{\mathit i=1}^{\mathit N^2}{({\mathit x}_{\mathit i}-\stackrel{-}{\mathit x})}^{2}\sum\:_{\mathit i=1}^{\mathit N^2}{({y}_{\mathit i}-\stackrel{-}{\mathit y})}^{2}}}$$

12 $$\:\stackrel{-}{\mathit x}=\frac{1}{\mathit N^2}\sum\:_{\mathit i=1}^{\mathit N^2}{\mathit x}_{\mathit i}\:\:,\text{ }\:\:\:\:\:\:\:\:\:\:\:\:\stackrel{-}{\mathit y}=\frac{1}{\mathit N^2}\sum\:_{\mathit i=1}^{\mathit N^2}{\mathit y}_{\mathit i}$$

**Table 5:** Correlation coefficient values of different plain images and their corresponding encrypted images.

Encrypted image *CR* = 0.4	Correlation coefficients					
	H		V		D	
	Encrypted	Original	Encrypted	Original	Encrypted	Original
Tank	0.0108	0.9459	0.0123	0.9329	0.0087	0.9020
Boat	0.0133	0.9378	0.0112	0.9708	0.0102	0.9218
Couple	0.0119	0.9363	0.0132	0.8934	0.0083	0.8569
Peppers R G B	0.0169 −0.0211 −0.0024	0.9621 0.9816 0.9668	0.0209 0.0299 0.0067	0.9669 0.9814 0.9691	0.0049 −0.0019 −0.0071	0.9572 0.9632 0.9510
Baboon R G B	−0.0048 −0.0146 0.0058	0.9164 0.8661 0.9070	0.0038 −0.0155 −0.0129	0.8626 0.7555 0.8753	−0.0099 −0.0076 −0.0168	0.8563 0.7319 0.8454
F-16 R G B	0.0086 0.0109 0.0186	0.9738 0.9690 0.9649	0.0343 −0.0054 −0.0174	0.9592 0.9687 0.9372	0.0059 0.0023 −0.0179	0.9398 0.9360 0.9225

The *CC* values of the encrypted and original images are calculated by randomly selecting 5000 pairs of nearby pixels in each direction. Table 5 gives a comparison of the *CC* values of the original and encrypted images. The *CC* values of the original images are very close to 1, indicating a strong correlation between adjacent pixels. In contrast, the *CC* values of the encrypted images are extremely low and can be considered negligible. This significant reduction in correlation demonstrates that the proposed framework effectively disrupts the statistical properties of the original images, and it is therefore resistant to statistical attacks. Moreover, compared with the schemes reported in^34^, the proposed framework achieves lower *CC* values for the encrypted images, further confirming its superior resistance to statistical attacks. Table 6 displays the pixel distributions of the original “Boat” image and the encrypted “Boat” image in all three directions. The pixel distribution of the original image exhibits a distinct pattern, whereas the pixel distribution of the encrypted image is uniform in all three directions.

**Table 6:**
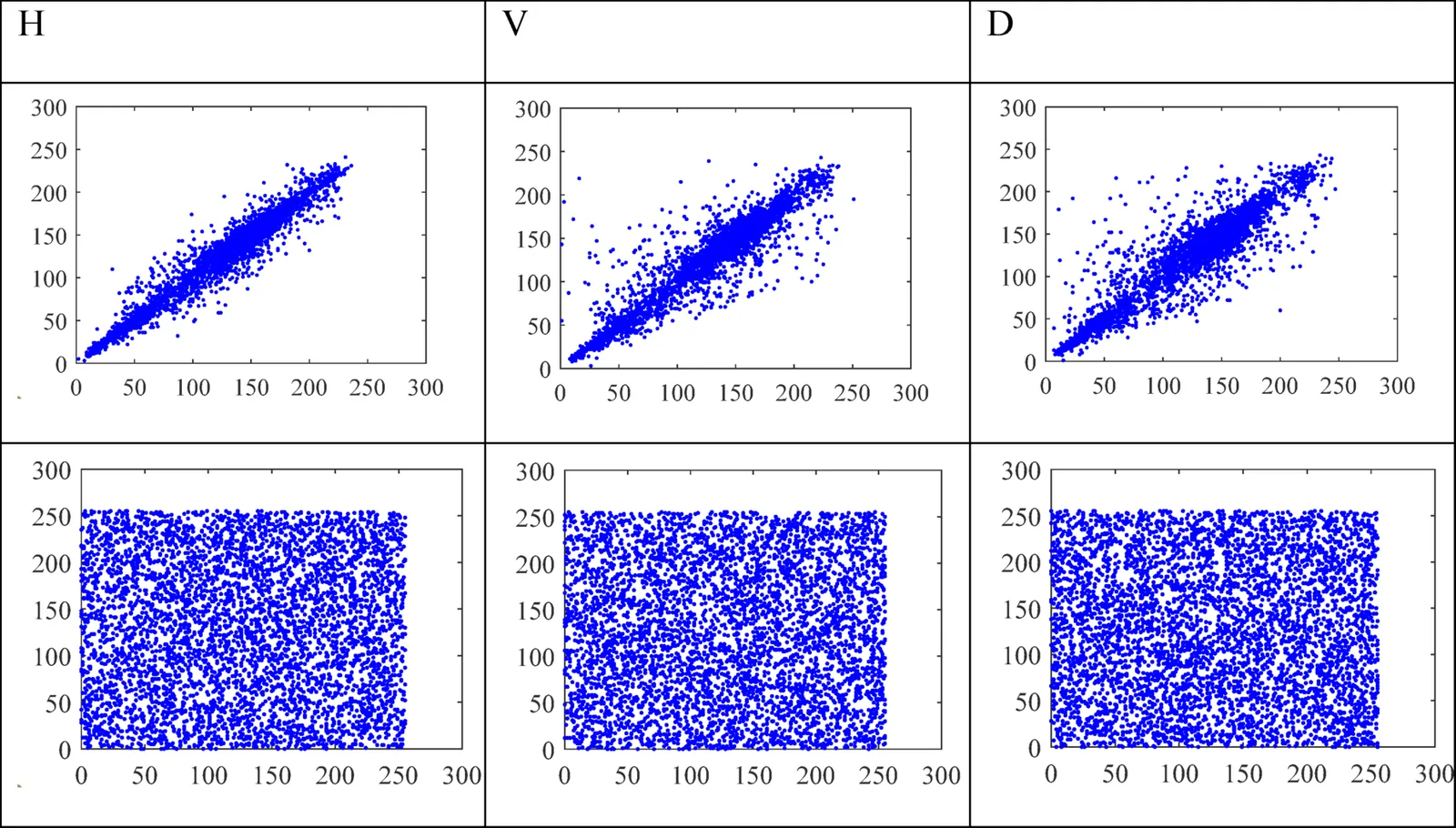
Horizontal, vertical, and diagonal correlation test results for the plain “Boat” image and the encrypted “Boat” image.

3. **Entropy test**

The information entropy (*E*) is a statistical measure of how uniformly pixel values are distributed. It is used to determine how well the encryption system works.

13 $$\mathit{E} = - \sum_{\mathit i=0}^{255} \mathit{P}_i \, \log_2 \mathit{P}_i$$

**Table 7:** Information entropy values of different plain images and their corresponding encrypted images.

Image	Plain image	Encrypted image
Tank	5.4957	7.9982
Boat	7.1914	7.9983
Couple	7.2010	7.9983
Peppers (R/G/B)	7.3388/7.4963/7.0583	7.9984/7.9981/7.9983
Baboon (R/G/B)	7.7067/7.4744/7.7522	7.9984/7.9983/7.9981
F-16 (R/G/B)	6.7178/6.7990/6.2138	7.9984/7.9982/7.9981

$\:\mathit {P}_{\mathit i}$ presents the probability of occurrence of the gray value $\:\mathit i$. For a gray image, the maximum theoretical information entropy is 8. An effective encryption technique should bring the encrypted image information entropy close to its theoretical value. For color images, entropy is calculated separately for the Red (R), Green (G), and Blue (B) channels. Ideally, the entropy values of the encrypted RGB channels should also be close to 8, indicating a uniform distribution of pixel intensities in each channel. The encrypted image should generally have higher information entropy than the original image entropy. Its entropy needs to be close to 8, indicating that its distribution is more uniform. As shown in Table 7, the entropy values of the encrypted images and the RGB channels for colour images are significantly higher than those of the original images and are very close to 8. This demonstrates that the encrypted images exhibit higher degrees of randomness and unpredictability.

Therefore, the proposed encryption framework effectively increases the entropy of images, making it resistant to entropy-based attacks.

4. **NIST randomness test**

NIST has presented a standard set of randomness assumptions^32^ to evaluate the effectiveness of encryption techniques. When the $\:\mathit p$ -value for a test exceeds the specified confidence level, we can conclude that the tested sequence is random with the corresponding confidence level. In this study, we perform the randomness test on the encrypted images, and the results are presented in Table 8, which shows that all tests pass with a confidence level of 0.01.

**Table 8:** National Institute of Standards and Technology (NIST) test for images.

NIST test	Tank *p*-value	Boat *p*-value	Couple *p*-value	Peppers *p*-value	Baboon *P*-value	F-16 *p*-value	Results
Frequency	0.534146	0.534146	0.739918	0.534146	0.739918	0.213309	Pass
Block Frequency	0.911413	0.911413	0.350485	0.534146	0.534146	0.017912	Pass
CumulativeSums-I	0.739918	0.350485	0.739918	0.534146	0.350485	0.213309	Pass
CumulativeSums -II	0.122325	0.739918	0.534146	0.350485	0.534146	0.350485	Pass
Runs	0.350485	0.008879	0.122325	0.534146	0.739918	0.213309	Pass
Longest Run	0.991468	0.911413	0.739918	0.213309	0.534146	0.911413	Pass
Rank	0.911413	0.213309	0.350485	0.350485	0.066882	0.534146	Pass
FFT	0.991468	0.350485	0.350485	0.534146	0.739918	0.350485	Pass
Non-overlapping Template	0.739918	0.534146	[0.350485–0.991468]	0.350485	[0.017912–0.991468]	0.534146	Pass
Overlapping Template	0.534146	0.739918	0.122325	0.739918	0.534146	0.534146	Pass
Approximate Entropy	0.534146	0.350485	0.017912	0.534146	0.739918	0.534146	Pass
Serial-I	0.350485	0.350485	0.534146	0.739918	0.534146	0.350485	Pass
Serial-II	0.122325	0.911413	0.213309	0.534146	0.534146	0.122325	Pass
Linear Complexity	0.534146	0.066882	0.534146	0.911413	0.350485	0.534146	Pass

It is important to note that the reported results are based on evaluations performed on a limited number of sequences generated from two or three test images. Therefore, the “Pass” decision reflects that each individual sequence meets the significance levels defined by the NIST tests rather than representing a statistical pass rate derived from a large set of sequences.

5. **Texture analysis**

A high-quality encryption algorithm should generate a pseudo-random cipher image with a uniform Grey-Level Co-occurrence Matrix (GLCM). To evaluate the texture characteristics of the encrypted image, second-order statistical features derived from the GLCM are analyzed. These features include contrast, correlation, energy, and homogeneity.

The results presented in Table 9 show that the proposed framework significantly increases the contrast while reducing correlation, energy, and homogeneity in the encrypted images. This indicates that the encryption process effectively disrupts the inherent texture structure of the original image, resulting in a random-like distribution of pixel intensities.

According to^33^, an attacker observing the CS measurements can capture only the signal energy information. Therefore, if the captured (cipher) image maintains approximately constant energy while concealing structural information, the CS-encrypted image can be considered to meet the requirements of perfect encryption.

**Table 9:** Second-order texture analysis statistics of encrypted images.

Image	Contrast	Correlation	Energy	Homogeneity
Tank	10.5120	0.0011	0.0156	0.3905
Boat	10.5498	0.0014	0.0156	0.3887
Couple	10.5383	0.0030	0.0156	0.3897
Peppers R G B	10.4121 10.5411 10.4695	0.0076 −0.0047 −0.0005	0.0156 0.0156 0.0156	0.3903 0.3892 0.3887
Baboon R G B	10.4402 10.5609 10.4699	0.0050 −0.0055 −0.0001	0.0156 0.0156 0.0156	0.3910 0.3892 0.3891
F-16 R G B	10.4361 10.4925 10.4621	0.0071 0.00003 −0.0007	0.0156 0.0156 0.0156	0.3913 0.3901 0.3896

### Differential attack analysis

The security level of an image encryption algorithm can be evaluated through its diffusion performance. Differential attack analysis is commonly used to assess this property. The resistance of the proposed framework to differential attacks depends on how a slight modification in the plain image affects the corresponding encrypted image.

To measure this effect, two metrics are used: NPCR and UACI. These metrics quantify the difference between two encrypted images generated from two plain images that differ by only one pixel.

Let $\bf \:{\mathit {I}}_{1}$ be the encrypted image corresponding to the original plain image, and $\bf \:{\mathit{I}}_{2}$ be the encrypted image corresponding to the plain image in which a single pixel has been modified. The NPCR and UACI values are then computed to evaluate the impact of this small change on the cipher image, where* NM *is the number of encrypted image pixels.

14 $$\:\mathrm{NPCR}\:=\frac{1} {\mathit NM}\sum\:_{i=1}^{\mathit N}\sum\:_{j=1}^{\mathit M}D(\mathit i,\mathit j)\times 100\mathrm{\%}\:\:\:$$

15 $$\:D\left(i,j\right)=\left\{\begin{array}{c}0 \quad\quad {{\mathit I}}_{1}\:\left(\mathit{i},\:\mathit{j}\right)\ne\:{{\mathit I}}_{2}\:(i,\:j)\:\\\: 1 \quad \quad {{\mathit I}}_{1}\:\left(\mathit{i},\:\mathit{j}\right)={{\mathit I}}_{2}\:(i,\:j)\end{array}\right.$$

16 $$\:\mathrm{UACI}=\frac{1}{\mathit NM}\sum\:_{\mathit i=1}^{\mathit N}\sum\:_{\mathit j=1}^{\mathit M}\frac{{{\mathit I}}_{1}\:\left(\mathit{i},\:\mathit{j}\right)-{{\mathit I}}_{2}\:(\mathit{i},\:\mathit{j})}{255}\:\times 100\mathrm{\%}\:\:$$

**Table 10:** NPCR and UACI values for the encrypted image test.

Image	NCPR %	UACI %
Tank	99.596|	33.522
Boat	99.596	33.348
Couple	99.596	33.396
Peppers	99.635	33.476
Baboon	99.598	33.474
F-16	99.611	33.428

The theoretical values of NPCR and UACI for an ideal encryption scheme are 99.6094% and 33.4635%, respectively. Table 10 shows that the obtained NPCR and UACI values are very close to these theoretical values. This indicates that the proposed encryption framework exhibits strong diffusion characteristics and is highly resistant to differential attacks.

### Key sensitivity analysis

**Fig. 5:**
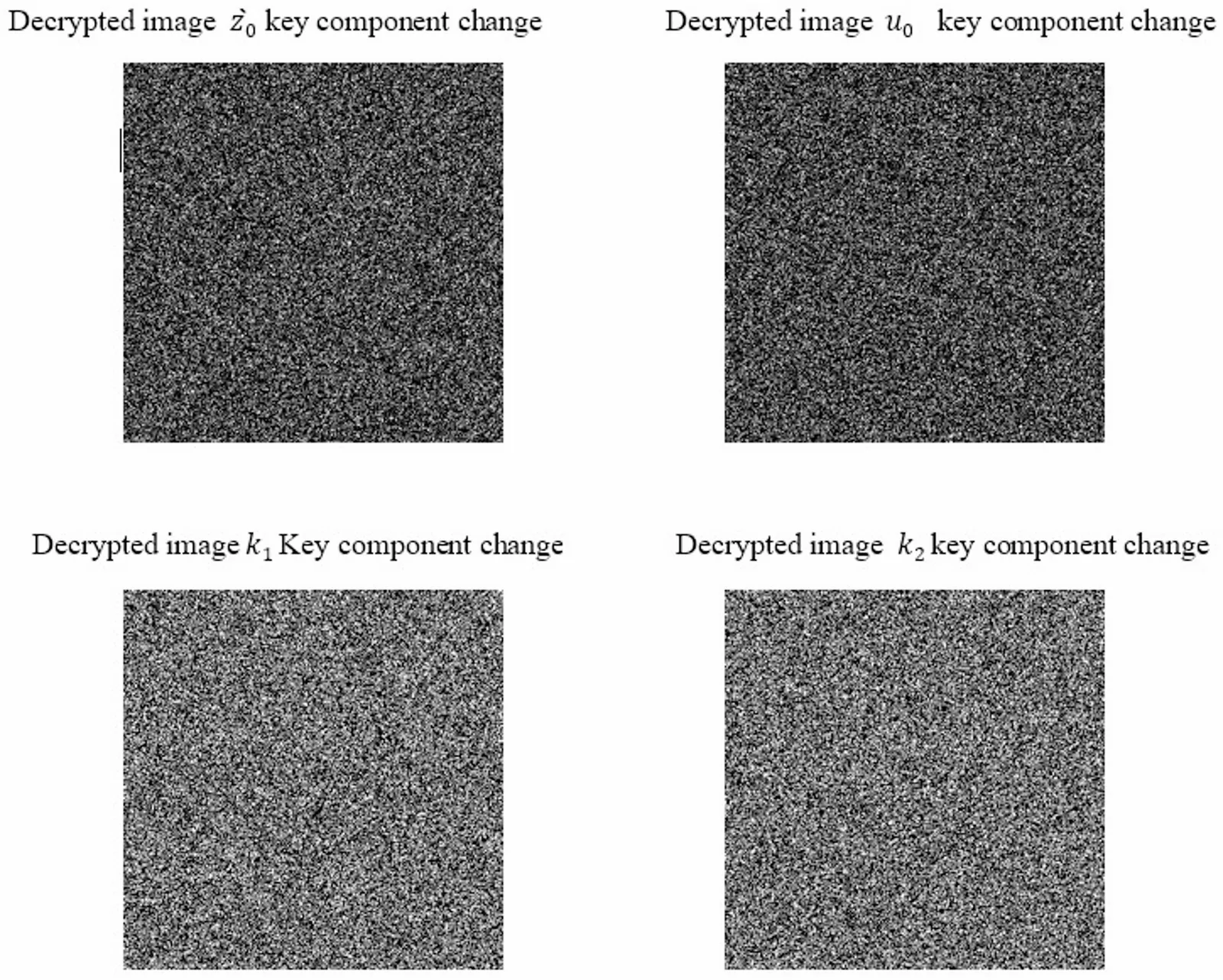
Decrypted images with modified key components.

To resist related-key attacks, an encryption scheme must exhibit high sensitivity to changes in the encryption key. Both the encryption and decryption processes should be strongly dependent on the key. To evaluate the key sensitivity of the proposed encryption process, the plain image is first encrypted using the correct keys giving a single encrypted image, and then the encrypted image is decrypted using a slightly modified key component, as the key generally has two parts and each part contains two components. The visual results of decryption, obtained after introducing very small changes on the order of $\:\left({10}^{-15}\right)$ in the key components are presented in Fig. 5. These results demonstrate that the decryption process is extremely sensitive to the keys used.

For visual comparison, the original image is encrypted and then decrypted after introducing a slight change in one of the encryption key components. A random set of key components (0.3, 0.3, 0.98, and 2.3) is used to encrypt and decrypt the images shown in Fig. 3, where the “Boat” image is selected as the test image. Using the correct keys in the decryption process, the framework successfully reconstructs the original plain image, as shown in Fig. 3. However, when a very small change is introduced in one of the key components, such as (0.300000000000001, 0.3, 0.98, and 2.3), the decryption process fails to recover the original image.

Figure 5 demonstrates that even when the decryption key components differ by as little as $\:{10}^{-15}$, the original image cannot be correctly recovered, resulting in a loss of vital information. This behavior highlights the strong sensitivity of the proposed framework to related-key attacks.

Table 11 presents the NPCR, UACI and correlation values between encrypted images when a slight change is introduced in one key component. Two keys $\:( \:{u}_{0}\:,\text { and } \acute{\mathit{z}_{0}})$ are used to control the measurement matrix generation, while the other two keys ($\:{\mathit k}_{1},\text{and }{\mathit k}_{2}$) control the random keystream generator used for the bitwise XOR operation. In the experiment, only one key component is modified with a very small perturbation in the range of $\:{10}^{-10}$, while the remaining key components remain unchanged. The obtained NPCR, UACI, and correlation values are close to their theoretical values, demonstrating that the proposed framework achieves high sensitivity to related-key attacks. Simulation results also indicate that changes in $\:{\mathit k}_{1}$, and $\:{\mathit k}_{2}\:$ produce similar effects; therefore, they are presented in a single column, reflecting their equal impact on the security of the random keystream generation.

**Table 11:** NPCR, UACI, and correlation values between encrypted images with slightly different key components.

Image	Correlation			NPCR%			UACR%		
	$\:\varDelta\:{\it{k}}_{1}\text {, }\varDelta\:{\it{k}}_{2}$	$\:\varDelta\:{\boldsymbol{z}}_{0}$	$\:\varDelta\:{\it{u}}_{0}$	$\:\varDelta\:{\it{k}}_{1}\text {, }\varDelta\:{\it{k}}_{2}$	$\:\varDelta\:{\boldsymbol{z}}_{0}$	$\:\varDelta\:{\it{u}}_{0}$	$\:\varDelta\:{\it{k}}_{1}\text {, }\varDelta\:{\it{k}}_{2}$	$\:\varDelta\:{\boldsymbol{z}}_{0}$	$\:\varDelta\:{\mathit {u}}_{0}$
Tank	0.0017	−0.0012	−0.0003	99.596	99.637	99.624	33.464	33.440	33. 538
Boat	0.0020	−0.0021	0.0004	99.623	99.587	99.609	33.429	33.418	33.459
Couple	0.0034	0.0022	0.0041	99.563	99.5998	99.614	33.373	33.418	33.464
Peppers	−0.0019	−0.0018	−0.0004	99,620	99.620	99.618	33.487	33.443	33.465
Baboon	−0.0025	−0.0025	−0.0016	99.620	99.612	99.627	33.508	33.484	33.499
F-16	0.0011	0.0007	−0.0008	99.611	99.587	99.605	33.428	33.423	33.475

The results confirm that the proposed encryption framework achieves strong security and robustness, making it suitable for secure image transmission in hostile environments.

### Key space analysis

The most basic form of cryptanalysis is a brute-force attack, in which an attacker attempts to discover the correct encryption key by exhaustively searching the entire key space. To resist such attacks, a secure encryption scheme must possess a sufficiently large key space, typically greater than 10^30^.

The proposed encryption framework has four secret keys: two keys are used for measurement matrix generation, while the other two keys control the random keystream generation process. Assuming the computational precision of each key is 10^− 14^, the total key space of the proposed framework can be calculated as $\:{\left({10}^{14}\right)}^{4}={10}^{56}$.

This large key space makes the proposed framework highly resistant to brute-force attacks. Even if a powerful attacker uses a conventional computing system capable of testing $\:{10}^{12}$ keys per second, it would require more than $\:{10}^{36}$ years to test the entire key space.

Furthermore, the proposed framework exhibits high sensitivity to the secret keys during both encryption and decryption processes. Even a very small change in any key results in completely different ciphertexts or unsuccessful decryption, thereby significantly enhancing the cryptographic security of the system.

### Time analysis

Real-time image processing in WSNs demands efficient encryption schemes capable of fast execution. Table 12 presents the average execution times for the encryption process, measured after twenty simulation runs for different images at varying *CR*s. The decryption time is not considered here, as the BS can typically be equipped with more powerful computational capabilities and parallel processing systems.

**Table 12:** Real-time image encryption times of different images with different *CR*s in sec.

Image/(*CR*)	0.15%	0.25%	0.35%	0.45%	0.55%	0.65%	0.75%
Peppers	0.6916	1.0387	1.3665	1.6989	2.0696	2.3857	2.7262
Pirate	0.6858	1.0308	1.3498	1.7132	2.0592	2.4156	2.7414
Cameraman	0.6909	1.0424	1.3453	1.7072	2.0504	2.4307	2.7524
Peppers	1.1278	1.8459	2.6024	3.3593	4.1689	5.1418	6.1198
Baboon	1.1586	2.0112	2.7998	3.5783	4.3636	5.1689	5.9242
F-16	1.1379	1.8917	2.7473	3.5477	4.1558	4.8224	5.8643

The images in the table include both grayscale and color images, with color images generally requiring more processing due to the increased data volume.

The linear computational complexity of $\:O\left(\mathit MN\right)$, combined with the use of lightweight arithmetic and logical operations, ensures that the proposed framework is suitable for real-time image processing in resource-constrained environments, such as WSNs and edge devices.

### Comparative analysis

To evaluate security and performance of the proposed framework, a comparative analysis was conducted using the Boat image. As shown in Table 13, the proposed framework achieves higher PSNR values than those of other schemes^7,35,36^.

**Table 13:** Encryption efficiency for different encryption schemes.

Scheme (*CR* = 0.35%)	PSNR	UACI %	NPCR %
Proposed	34.31	33.41	99.61
^7^	31.15	33.45	99.56
^35^	31.62	33.48	99.60
^36^	28.95	32.71	99.52

The proposed framework is further evaluated using another image of size $\:256\times\:256$. As shown in Table 14, the PSNR value of the proposed framework demonstrates improved performance.

**Table 14:** PSNR comparison.

Scheme (*CR *= 0.5%)	PSNR (dB)
Proposed	36.29
^22^	29.82
^37^	30.25
^38^	< 26
^14^	29.2337

Compared with recent 2024–2025 papers on image encryption, and compression–encryption schemes, the proposed framework demonstrates clear advantages in Table 15. Although hyperchaotic CS-based schemes^14,15^ provide strong security, they suffer from high computational complexity and parameter-management overhead. Lightweight chaos-only schemes^20^ reduce complexity but lack inherent compression. DNA-based IoT encryption schemes^21^ achieve high diffusion but do not integrate CS.

In contrast, the proposed framework achieves simultaneous compression and encryption, superior reconstruction quality (PSNR = 36.39 dB at *CR* = 0.25), strong diffusion metrics (NPCR ≈ 99.6%, UACI ≈ 33.5%), and reduced key-storage requirements, making it more suitable for real-time and resource-constrained WSN environments.

**Table 15:** Comparison with recent 2024–2025 papers on image compression–encryption schemes.

Scheme	Year	Method	*CR*	PSNR (dB)	NPCR (%)	UACI (%)	Complexity
Liu et al.^14^	2024	2D CS + Hyperchaos	0.25	29.23	99.60	33.48	High
Yao et al.^15^	2025	CS + 4D Hyperchaos	0.25	~ 31.5	99.61	33.51	Very High
Lai & Hua^21^	2025	DNA + Hyperchaos	–	–	99.62	33.54	High
Odeh et al.^20^	2025	Tent-Map Chaos	–	–	99.58	33.41	Low
**Proposed**	**----**	**BCS + DNA + XOR**	**0.25**	**36.39**	**99.61**	**33.54**	**Low–Moderate**

### Complexity and real-time application

1. **Complexity of encryption at sensor node**:

The encryption process consists of four main stages. For an image of size $\:\mathit N\times\:\mathit N$ divided into blocks of size $\:\mathit b\times\:\mathit b$, the total number of blocks is $\it \:n\:=\:{\left(\frac{\it N}{\it b}\right)}^{2}$.

**Block Compressive Sensing (BCS)**.

In BCS, multiplying a matrix $\:\mathbf {\Phi\:}$ by a vector $\:\mathbf{x}$ is of $\it \:O\left(M\:{b}^{2}\right)$.

- Each block calculation: ${\mathit \:O}\left({\mathit M{\:b}^{2}}\right)={\mathit \:O}({\mathit CR\times{\:b}^{4}})$, where $\it \:M\:=\:CR\times{b}^{2}.$
- **Total Complexity**: $${\it \:n\times O}\left(\it CR\:\times\:\:{b}^{4}\right)={\it O}\left(\:{\left(\frac{N}{b}\right)}^{2}\times\:\it CR\:\times\:\:{b}^{4}\right)={\it O}(\:{\it CR\:\times\:\:{N}^{2}\times\:{b}^{2}})$$.

Assuming a fixed block size, this simplifies to:

$${\it \:O}(\:CR\:\times\:\:{N}^{2})$$

- *Memory Optimization*: The measurement matrix $\:\boldsymbol{\Phi\:}$ is generated using the Mersenne Twister (MT19937) PRNG. Only the seed and update logic are stored, resulting in ${\it \:O}\left(1\right)$ memory complexity for matrix storage.
- ** DNA Encoding, and Bit-Level XOR**
- **DNA Encoding**: Every pixel is converted into four DNA bases using a lookup table. This operation is linear in complexity with $\it \:O\left({N}^{2}\right).$
- **Stream Cipher (XOR)**: Generating the keystream and applying the XOR is a bitwise operation, which has a complexity of ${\it \:O}({\it CR\:\times\:\:{\it N}^{2}})$.

**Overall Node Complexity**: Combining all operations, the overall complexity for encryption at the sensor node is of ${\it \:\:O}({\mathit CR\:\times\:\:{\mathit N}^{2}})$.

Since *CR* is typically less than 1 (usually between 0.25 and 0.4), the proposed encryption framework is computationally lightweight, making it well-suited for resource-constrained sensor nodes when compared to more computationally intensive methods, such as heavy transform-based compression or public-key encryption schemes.

2. **Complexity of Decryption at BS**.

At the BS, image reconstruction is performed using Bayesian Compressive Sensing-Relevance Vector Machine (BCS-RVM), combined with the Dual-Tree Discrete Wavelet Transform (DT-DWT). The DT-DWT provides a sparse representation of the image features, while the RVM serves as a Bayesian sparse recovery framework. This framework estimates both the signal coefficients and their posterior distributions, ensuring efficient reconstruction of the encrypted image.

**Computational Characteristics**:

Unlike standard convex $\:{l}_{1\:}$-minimization optimization methods such as LASSO, the BCS-RVM employs an automatic relevance determination mechanism that promotes sparsity. This mechanism often achieves accurate reconstruction using fewer measurements. Although RVM typically incurs higher computational complexity due to iterative Bayesian inference, this overhead is manageable at the BS.

It is assumed that the BS possesses powerful computational resources and parallel processing capabilities, which allow for the efficient execution of the decryption and reconstruction processes without significantly impacting system latency.

3. **Real-Time Application and WSN Deployment**.

The proposed framework is well-suited for real-time applications, as demonstrated by the timing analysis in Table 12. For a 512 × 512 image, the total encryption time for gray images ranges from approximately 0.6 s to 2.7 s, depending on the *CR*.

4. **Suitability for WSN s and IoT Environments**.

The framework is particularly effective in WSNs and IoT environments for the following reasons:

- **Memory Efficiency**: The measurement matrix $\:\mathbf{\Phi\:}$ is generated on-the-fly using a modified Tent map, which eliminates the need to store a large sensing matrix. Without this optimization, storing the matrix would require $\:\it M\times\:{\it b}^{\it 2}\:\:$ floating-point entries per block. Instead, only the initial parameters of the chaotic map need to be stored, significantly reducing memory usage.
- **Energy Conservation**: In WSNs, radio transmission is the primary source of energy consumption. By reducing the amount of transmitted data through CS (e.g., transmitting only 25% of the original data at *CR* = 0.25), the energy saved during wireless transmission outweighs the computational cost of lightweight operations such as XOR and DNA encoding.
- **Parallelism**: The BCS technique allows each image block to be processed, independently. This inherent parallelism makes the proposed framework suitable for efficient implementation on hardware platforms with Single Instruction, Multiple Data (SIMD) capabilities or multi-core microcontrollers. Such parallel processing reduces encryption latency and further enhances real-time performance.

### Resource, channel, and energy limitations

#### Hardware resource constraints

In real-world deployments, sensor nodes such as TelosB, MICAz, or ESP32 are subject to significant limitations in terms of RAM and computational capacity. Although BCS is adopted to reduce memory overhead, the simulation environment assumes that both image blocks and the sensing matrix $\:\mathbf{\Phi\:}$ can be held in memory, simultaneously.

For instance, a 32 × 32 image block, stored in 32-bit floating-point format, requires approximately 4 KB of memory. When combined with additional buffers and DNA lookup tables, this memory requirement may exceed the available SRAM of low-end microcontrollers.

To address this limitation, a memory-efficient streaming implementation has been proposed. By generating the measurement matrix $\:\mathbf{\Phi\:}$ row-by-row, on-the-fly, the node avoids the $\it \:O\left(M\:{b}^{2}\right)$ RAM overhead associated with storing the full sensing matrix. As a result, the working memory is limited to a single image block and a single measurement vector, ensuring compatibility with the typical SRAM constraints of IoT devices (e.g., 16–64 KB).

Additionally, many WSN platforms lack a hardware Floating-Point Unit (FPU), and standard CS operations involve floating-point arithmetic. Therefore, validating the proposed framework using fixed-point arithmetic is crucial for practical deployment on such devices.

#### Impact of Unreliable Wireless Channels

While most simulations assume ideal transmission conditions, real-world WSN environments are subject to interference, multipath fading, and environmental noise.

- **Packet Loss and Bit Errors**: Protocols like IEEE 802.15.4 are vulnerable to bit errors and packet loss. Since the proposed framework uses DNA encoding and bit-level XOR operations, even a single bit error in the ciphertext can result in error propagation during DNA decoding, potentially corrupting the entire decrypted block.
- **Fading and Shadowing**: Wireless signal fluctuations may cause packet drops, leading to missing measurements. While CS is generally robust to partial data loss, experimental evaluation is needed to determine the tolerance of the DNA-based decryption layer under such conditions.

#### Energy Consumption Trade-offs

While the “**Simulation Results and Discussion**” section reveals that the proposed framework increases network lifetime, a thorough validation through energy-based measurements is required.

- **Computation versus Transmission**: It is essential to verify whether the energy savings achieved by reducing the volume of transmitted data (e.g., transmitting only 40% of the data at *CR* = 0.4) outweigh the energy consumed by additional computations such as chaotic map generation, MT19937 keystream generation, DNA encoding, and XOR operations.
- **Real-Time Power Profiling**: Direct measurement of energy consumption using hardware tools (e.g., oscilloscopes, power monitors) or experimental platforms such as **FIT IoT-LAB** would provide quantitative evidence of the system energy efficiency by capturing the real-time current drawn during the encryption process.

.

## Conclusion

This work presented a privacy-preserving and efficient framework for image transmission in WSNs, combining BCS, DNA encoding, and bit-level XOR to simultaneously compress and encrypt data. By generating the measurement matrix $\:\mathbf{\Phi\:}$. On-the-fly use of a chaotic Tent map and employment of MT19937 for keystream generation, the framework reduces memory requirements, strengthens security, and separates randomness sources.

Simulation results demonstrate high reconstruction quality, resilience to statistical attacks, and a significant reduction in the transmitted data volume, emphasizing the framework suitability for resource-constrained sensor nodes.

Future work will focus on transitioning this framework to practical deployment. Key directions include:

- Hardware-aware implementation on platforms such as ESP32 and ARM Cortex-M, optimizing matrix operations and employing fixed-point arithmetics.
- Empirical energy profiling in live WSNs.
- Robust handling of packet loss and channel noise through error correction codes and adaptive BCS recovery.
- Algorithmic improvements, such as hyper-chaotic integration, adaptive compression, and deep learning-based reconstruction at the BS.

These efforts aim to enable secure, high-performance, and energy-efficient sensing architectures for next-generation IoT networks.

### Practical Limitations and Future Work

A primary limitation of the proposed framework is its reliance on floating-point arithmetic for the modified Tent map and CS matrix operations. Many low-power WSN nodes (e.g., those based on 8-bit or 16-bit microcontrollers) lack dedicated Floating-Point Units (FPUs), requiring software emulation of these operations. This emulation increases instruction cycles and energy consumption, potentially offsetting the compression benefits.

To mitigate these issues, future work will focus on porting the framework to a fixed-point representation. This will involve scaling chaotic map parameters and measurement matrix $\:\mathbf{\Phi\:}$ into integer formats (e.g., Q15 or Q31 standards). By replacing floating-point multiplication with integer bit shifts and additions, we expect to reduce CPU cycles significantly—potentially improving execution speed by a factor of 3 to 5 on standard IoT hardware, without compromising reconstruction quality, PSNR or the chaotic properties of the encryption keys.

## Data Availability

The data used to support the findings of this study are available from the first or corresponding author upon request.
